# Supplementary material for: Knowledge and Awareness of Risk Factors for HIV Infection and about HIV Testing among Medical Students in Warsaw
Source: Viruses. 2024 Sep 15;16(9):1470. doi: 10.3390/v16091470 (PMC11437482; doi:10.3390/v16091470)
Supplement: Supplementary file 1 [file viruses-16-01470-s001.zip › viruses-3193653-supplementary.pdf]

Questionnaire for the survey

1. Gender
    a. Male
    b. Female
    c. Other
2. Age in years (open question)
3. Country of origin (open question)
4. Faculty
    a. Medical analytics
    b. Audiophonology with hearing care
    c. Dietetics
    d. Electroradiology
    e. Pharmacy
    f. Physiotherapy
    g. Dental hygiene
    h. Medicine
    i. Dental medicine
    j. General and clinical logopaedics
    k. Nursing
    l. Midwifery
    m. Emergency medicine
    n. Dental technology
    o. Toxicology with elements of forensic science
    p. Public health
    q. Other
5. Year of studies
    a. 1
    b. 2
    c. 3
    d. 4
    e. 5
    f. 6
6. Medical specialty you plan to practice in future (open question)
7. In terms of HIV which body fluids are contagious? (multiple-choice question)
    a. Bloof
    b. Semen
    c. Saliva
    d. Vaginal excretion
8. Choose diseases or conditions which DOES NOT require testing for HIV (multiple-choice question)
    a. Cervical cancer
    b. Syphilis
    c. Brain abcess

    d. Lung cancer
9. Can a person living with HIV and being on effective antiretroviral therapy (multiple-choice question)
    a. Be a blood donor
    b. Have unprotected sex with
    c. Have a natural birth
    d. Perform a medical profession
10. What is the risk of mother to child HIV transmission, if mother is not on treatment and no risk reduction intervention is used?
    a. 1%
    b. 10%
    c. 30%
    d. 70%
11. What is the risk of HIV transmission for unprotected vaginal sexual contact?
    a. 1/10000 contacts
    b. 1/100 contacts
    c. 1/10 contacts
    d. 0
12. What is the risk of HIV transmission for unprotected anal sexual contact?
    a. 3/10000 contacts
    b. 3/100 contacts
    c. 3/10 contacts
    d. 0
13. Do you personally know anyone who is living with HIV?
    a. Yes
    b. No
14. Have you ever had an HIV test?
    a. Yes
    b. No
15. Have you ever proposed HIV test to someone else?
    a. Yes
    b. No
16. Have you ever heard about HIV self-testing before?
    a. Yes
    b. No
17. Have you ever heard about U=U principle?
    a. Yes
    b. No
18. In your opinion who should get tested for HIV?
    a. Everyone with at least one risky behavior in past (eg. unprotected sex, injecting drugs)
    b. Only people with repeated risky behaviors
    c. Only people who are asymptomatic

    d. All children under 5 years old

19. You consult a person who had risky behavior a week ago. In the context of HIV what do you do?
    a. Prescribe post-exposure prophylaxis
    b. Postpone HIV testing until 3 weeks (since it is too early to test)
    c. Test for HIV, if negative you end the follow-up
    d. Test for HIV and if negative you recommend to repeat test after 6 weeks
20. Is self-test for HIV is negative, would you recommend to repeat it?
    a. No
    b. Yes, but only after next risky behavior
    c. Yes, after 48 hours
    d. Yes, after 12 weeks
